# Plant GARDEN: a portal website for cross-searching between different types of genomic and genetic resources in a wide variety of plant species

**DOI:** 10.1186/s12870-023-04392-8

**Published:** 2023-08-12

**Authors:** Hisako Ichihara, Manabu Yamada, Mitsuyo Kohara, Hideki Hirakawa, Andrea Ghelfi, Takuro Tamura, Akihiro Nakaya, Yasukazu Nakamura, Sachiko Shirasawa, Samatchaya Yamashita, Yosuke Toda, Daijiro Harada, Tsunakazu Fujishiro, Akiko Komaki, Jeffrey A. Fawcett, Eiji Sugihara, Satoshi Tabata, Sachiko N. Isobe

**Affiliations:** 1 Kazusa DNA Research Institute, Kisarazu, Chiba 292-0813 Japan; 2 Bioinformation and DDBJ Center, National Institute of Genetics, Mishima, Shizuoka 411-8540 Japan; 3 Research and Development Center for Precision Medicine, University of Tsukuba, Tsukuba, Ibaraki 305-8550 Japan; 4 Graduate School of Frontier Sciences, The University of Tokyo, Kashiwa, Chiba 277-0882 Japan; 5 Present Address: Chitose Bio Evolution Pte. Ltd. Kawasaki, Kanagawa, 213-0012 Japan; 6 Present Address: RIKEN iTHEMS, Wako, Saitama 351-0198 Japan; 7 Present Address: Division of Gene Regulation, Fujita Cancer Center, Research Promotion Headquarters, Fujita Health University School of Medicine, Toyoake, Aichi 470-1192 Japan

**Keywords:** Database, Plant, Genome, Gene, Marker, SNPs, Cross-search

## Abstract

**Background:**

Plant genome information is fundamental to plant research and development. Along with the increase in the number of published plant genomes, there is a need for an efficient system to retrieve various kinds of genome-related information from many plant species across plant kingdoms. Various plant databases have been developed, but no public database covers both genomic and genetic resources over a wide range of plant species.

**Main body:**

We have developed a plant genome portal site, Plant GARDEN (Genome And Resource Database Entry: https://plantgarden.jp/en/index), to provide diverse information related to plant genomics and genetics in divergent plant species. Elasticsearch is used as a search engine, and cross-keyword search across species is available. Web-based user interfaces (WUI) for PCs and tablet computers were independently developed to make data searches more convenient. Several types of data are stored in Plant GARDEN: reference genomes, gene sequences, PCR-based DNA markers, trait-linked DNA markers identified in genetic studies, SNPs, and in/dels on publicly available sequence read archives (SRAs). The data registered in Plant GARDEN as of March 2023 included 304 assembled genome sequences, 11,331,614 gene sequences, 419,132 DNA markers, 8,225 QTLs, and 5,934 SNP lists (gvcf files). In addition, we have re-annotated all the genes registered in Plant GARDEN by using a functional annotation tool, Hayai-Annotation, to compare the orthologous relationships among genes.

**Conclusion:**

The aim of Plant GARDEN is to provide plant genome information for use in the fields of plant science as well as for plant-based industries, education, and other relevant areas. Therefore, we have designed a WUI that allows a diverse range of users to access such information in an easy-to-understand manner. Plant GARDEN will eventually include a wide range of plant species for which genome sequences are assembled, and thus the number of plant species in the database will continue to expand. We anticipate that Plant GARDEN will promote the understanding of genomes and gene diversity by facilitating comparisons of the registered sequences.

**Supplementary Information:**

The online version contains supplementary material available at 10.1186/s12870-023-04392-8.

## Background

The growing area of plant research and development (R&D) is characterized by two major types of diversity: a diversity of species to be studied and a diversity of research communities, ranging from groups performing basic research to those involved in industrial applications. In particular, the fact that the number of plant species used for industrial purposes—including as food and energy raw materials—is much larger than the corresponding number of animal species used in industry, is a major factor in the great diversity of both species and communities targeted for R&D.

Plant genome information is fundamental to plant R&D. Next-generation sequencing (NGS) technologies, which were first used in the research field around 2010, have accelerated the amount of plant genome information generated worldwide. Especially in recent years, de novo assembly of genome sequences and resequencing of numerous varieties and lines have been conducted in many plant species [1]. For example, as of March 2023, the NCBI genome database (https://www.ncbi.nlm.nih.gov/genome/) had entries for 3,089 genomes for 1360 *Viridiplantae* species, 43 genomes for 20 *Rhodophyta* species, and eight genomes for 11 *Glaucophyta* species. This high availability of data has led many biologists and industries to accelerate the introduction of genome information in their research.

In conjunction with the development of NGS technologies, several plant genome databases covering a broad diversity of plant species have been developed, such as Phytozome [2], Ensembl Plants [3], GreenPhylDB [4], and PlabiPD (https://www.plabipd.de/). For example, in Phytozome, 147 species and 295 genomes were available in March 2023, with lists of genes showing high homology to the selected gene. On the other hand, at the same time, Ensembl Plants has registered 132 genomes derived from 108 species. Ensembl Plants also provides comparative genomic analyses that allow researchers to compare genetic features across different plant species. PlabiPD is a unique database of plant genomic information, providing graphical representations of the status of plant genome decoding using timelines or phylogenetic positions. It offers a visual representation of plant genome information that is not found in other plant genome databases. These databases provide comprehensive phylogenetic relationships of plant genes and species based on evolutionary information such as orthologs and paralogs. However, they do not provide information related to genetic studies, such as genome-wide SNP lists and DNA markers.

Comprehensive databases that provide the data needed for both genomic and genetic analyses, including genes, DNA markers, and trait-related loci along with the reference genome sequence, have been developed as family- or species-specific databases, such as Gramene for *Poaceae* [5], SGN (Sol Genomics Network) for *Solanaceae* [6], and LIS (Legume Information System) for *Fabaceae* [7]. However, these databases are only for plant species belonging to specific taxa and do not allow for sequence comparisons across species in the plant kingdom.

As the number of published plant genomes continues to increase, there is a need for an efficient system to retrieve various genome-related data from many plant species across plant kingdoms. The Plant GARDEN database (Genome And Resource Database Entry: https://plantgarden.jp) presented in this paper was developed as a portal website to provide genome-related information such as genomes and DNA markers of various plant species covering many families, as well as tools for comparative analysis of the information both within and between species. In addition, we have developed a web-based user interface (WUI) that enables a diverse range of users to access information in an easy-to-understand manner, in the anticipation that genome analysis will be routinely performed not only by R&D specialists but also in an educational context and other relevant areas.

## Construction and content

### Implementation

**Fig. 1:**
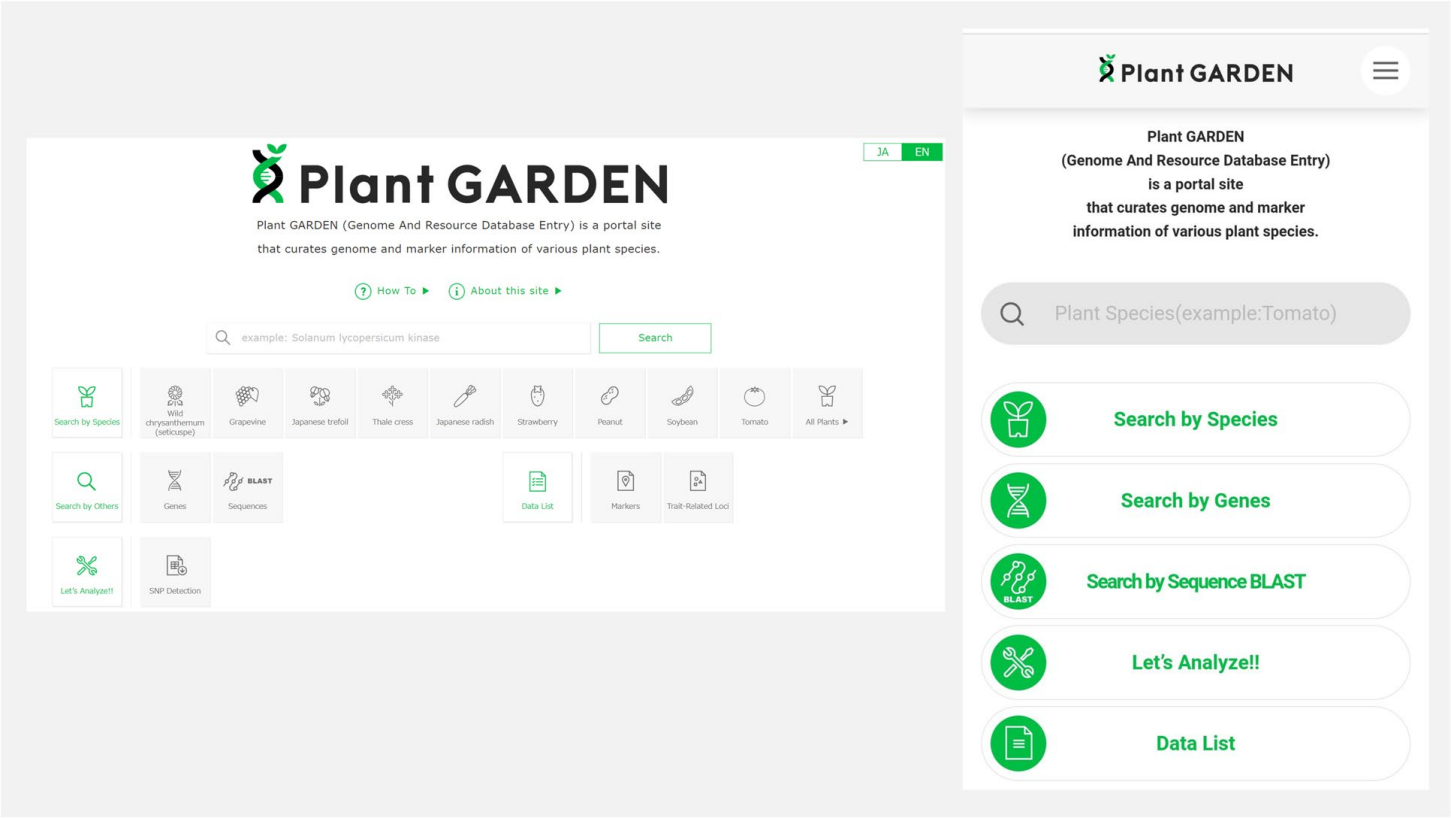
Plant GARDEN top page screen for the PC (left) and tablet (right) versions. Four or five major categories are displayed on the top page for different approaches to the content search

The web address of Plant GARDEN is https://plantgarden.jp. Plant GARDEN was developed in Apache (https://db.apache.org/), MySQL (https://www.mysql.com/), and PHP (https://www.php.net/) and built by XAMPP (https://www.apachefriends.org/) on a Linux server with RHEL (Rad Hat Enterprise Linux). The database for the contents was built using MariaDB (https://mariadb.org/), a MySQL-compatible database. A PHP framework, Laravel (https://laravel.com/), was used to construct the WUI. Different types of WUI were developed for display on personal computers (PCs) and tablet computers (hereinafter tablets) to make data searches more convenient (Fig. 1). The system automatically selects the appropriate version (PC or tablet) of the display based on the type of operating system used. Some pages, such as the browser, were developed only for the PC version but can also be accessed from the tablet version. The available languages are English and Japanese.

The data structure was designed in the REST (REpresentational State Transfer) style, and restful API is used to display the data in the tablet version. Since the PC version was developed early in the creation of Plant GARDEN, the data are displayed by direct query to the SQL database. A keyword search system named pges was developed for rapid full-text search in Plant GARDEN (Supplementary Fig. S1). Elasticsearch (https://www.elastic.co/) was used as the search engine, and an API server, crawler, and indexer were developed with perl scripts.

### Outline of the contents

**Fig. 2:**
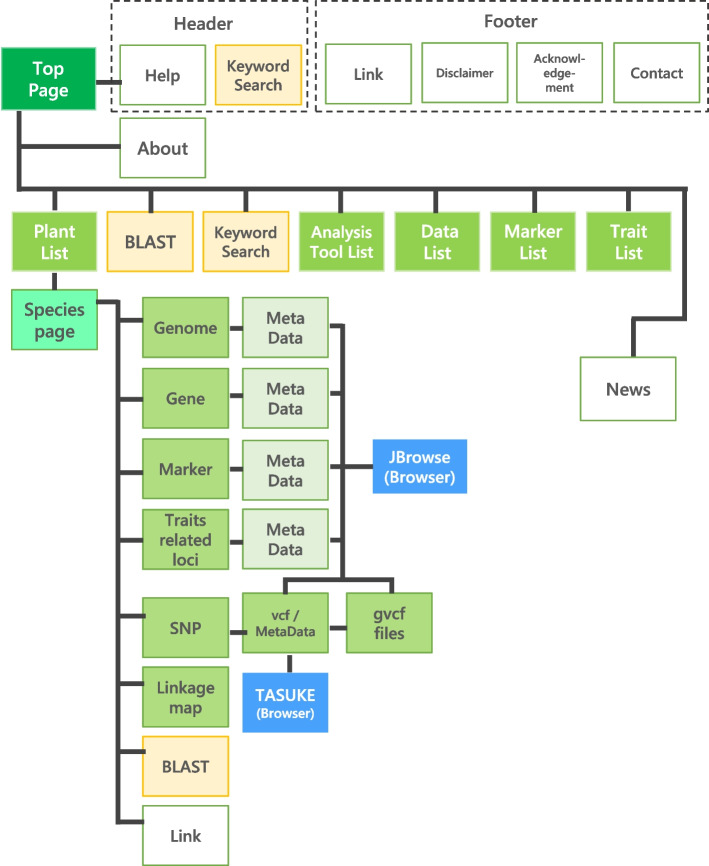
Systematic framework diagram of Plant GARDEN

Plant GARDEN will eventually include a wide range of plant species for which genome sequences are assembled, and thus the number of plant species in the database will continue to expand (Supplementary Fig. S2). At the end of each year, we report the number of data added that year on the News pages of Plant GARDEN. The database contains the following types of data: 1) assembled genome sequences, 2) gene sequences, 3) base variants (SNPs and in/dels) detected on SRAs (Sequence Read Archives) against an assembled genome, 4) PCR-based DNA markers, 5) trait-related loci identified by genetic studies, and 6) linkage maps (Fig. 2). The sequence and marker information were collected in Plant GARDEN by curating public data except for base variants on SRAs. The genus, family, and common name to which each stored plant species belongs are also provided as general information.

As of March 28, 2023, the available information included 304 genome sequences from 234 species, 11,331,614 gene sequences from 162 species, 419,132 DNA markers from 72 species, 8,255 trait-related loci from 28 species, linkage maps from 1 species, and variant information (gvcf) from 105 species, consisting of 5,934 data points (Table 1). The stored data are also listed in the Data List, which is accessible at the top page of Plant GARDEN. The files obtained from original sources, including genome and gene sequences and gene annotation files, can be downloaded from the download site on Plant GARDEN.

**Table 1:** Main data contents provided in Plant GARDEN (March 28, 2023)

Data type	Number of species	Number of data
Genome	234	304
Gene	162	11,331,614
Base variants on SRA (VCF)	106	108
Base variants on SRA (gVCF)	105	8,934
DNA marker	72	419,132
Trait-linked DNA marker (QTL)	28	8,255

### Assembled genome sequences

Most of the genome sequences in Plant GARDEN are collected from public databases available on the condition that they were assembled at the chromosome level, but several scaffold-level sequences were also registered in Plant GARDEN upon request from users. The source name and URL of the site providing the sequence data are shown on each genome sequence page, with the statuses of the assembled genomes and information on the source article along with contact information to reach the corresponding author (Supplementary Fig. S3). In cases in which the genome sequences were directly submitted to NCBI without being published in an article, the entry was designated as a "Direct submission" in the article information field. The quality evaluation of assembled genomes and gene sequences was performed by our team using BUSCOs [8].

### Functional re-annotation of genes associated with OrthoDB

In general, inferred genes on a genome are functionally annotated by the publisher of the genome sequence. The annotation criteria, databases used, and analysis items differ among publishers, which makes it difficult to compare orthologous relationships of genes based on the provided annotation information. Thus, we have re-annotated functions of all the genes registered in Plant GARDEN by using a functional annotation tool, Hayai-Annotation [9], based on KusakiDB (https://github.com/aghelfi/kusakiDB). KusakiDB is a database of orthologous genes in plants, consisting of sequence information derived from OrthoDB (https://www.orthodb.org/), UniProt (https://www.uniprot.org/), and RefSeq (https://www.ncbi.nlm.nih.gov/refseq/). Re-annotation of gene function is carried out on the gene sequence file (pep file) created by the publisher on the genome, and the gene sequence names are used as defined by the publisher. The re-annotated genes are associated with OG (Ortholog Group) IDs on OrthoDB to find ortholog gene sequences in other species (Supplementary Fig. S4). In addition, Hayai-Annotation detects the most similar gene from among the genes registered in UniProtKB (https://www.uniprot.org/help/uniprotkb) via KusakiDB, and estimates the GO, KEGG, Pfam, Interpro ID, EC, and TF families. If there are no similar gene sequences in UniProtKB, it refers to RefSeq. When referring to RefSeq, functional annotation is carried out using InterProscan (https://www.ebi.ac.uk/interpro/) for GO, Pfam, and Interpro ID.

### Base variants on SRA

Base variants on SRAs are unique contents generated by the Plant GARDEN project. We collected a maximum of 100 SRA sequences from either the NCBI database (https://www.ncbi.nlm.nih.gov/sra) or DDBJ (https://www.ddbj.nig.ac.jp/dra/index-e.html), prioritizing those with higher genome coverage and a greater use of common plant materials. The variants were identified by a combination of Bowtie2 [10] and SAMTools [11] for mapping and variant calls, respectively, or by an Illumina DRAGEN pipeline (Illumina, San Diego, CA, USA). When multiple reference genome sequence files were available for a species at the time of analysis, we selected a single sequence file based on assembly quality and the file's significance in the research community. A gVCF (genomic variant call format) file was created for each SRA file, and a VCF (variant call format) file was subsequently created by integrating all the gVCF files created in a species (Supplementary Fig. S5). VCF files provided the variant information in a specific format, making it difficult to understand the descriptions, especially for users who were not specialists in genetics. Therefore, we converted the polymorphic allele information in the vcf file to the bases with the letters A, G, T, and C and provided this information as a text table. Although we created most of the gvcf and vcf files in Plant GARDEN, Plant GARDEN also accepts the registration of publicly available vcf and gvcf files.

### PCR-based DNA markers

PCR-based DNA markers, categorized as SSR (simple sequence repeats), CAPS/dCAPS (cleaved amplified polymorphic sequences), SCARs (sequence characterized amplified regions), and others were collected by manual curation of published articles. The information on markers available in PGDBj [12] and the Kazusa Marker DataBase [13] were also registered in Plant GARDEN. Each reference genome in each species was selected on the basis of assembly quality and importance to the research community. Marker positions on the selected reference genomes were estimated by BLAST analysis of primer or target sequences of the markers with the default parameters and the '-task blastn-short' option. The following criteria were also used to determine the positions: 1) The maximum mismatch is equal to or less than 2 bp. 2) The distances between forward and reverse primers were within 5 Kb. If multiple hits were observed, the top hit result was selected, and the number of the hit positions was given in the comment column. Marker names, types, positions on the reference genome, and the name of the reference genome are shown in a marker list and marker details pages (Supplementary Fig. S6). Primer or target sequences and source manuscript information are also available on the marker details page.

### Trait-related loci

Trait-related loci identified by genetic studies, such as interval mapping or genome-wide association studies (GWAS), were also registered in Plant GARDEN by collecting the information with manual curation of the published articles. The trait types were classified as stress, fertility, yield, morphology/growth, quality/components, and others. The positions of the registered loci on the reference genome are graphically shown on the trait-related loci list page, and a locus list is shown under the graphical view (Supplementary Fig. S7A and B). The locus details page shows the locus name, trait name, trait category, analysis method, population name, population type, position on the genome estimated via nearest markers, position on the linkage map (if available in the source manuscript), and source manuscript (Supplementary Fig. S7C).

## Utility

### Search by species

Several processes are available for searching the contents in Plant GARDEN. We considered that searches by species would be the most frequently performed, because much plant R&D targets specific species. Therefore, various types of registered data are organized and aggregated on the species page set for each plant species.

**Fig. 3:**
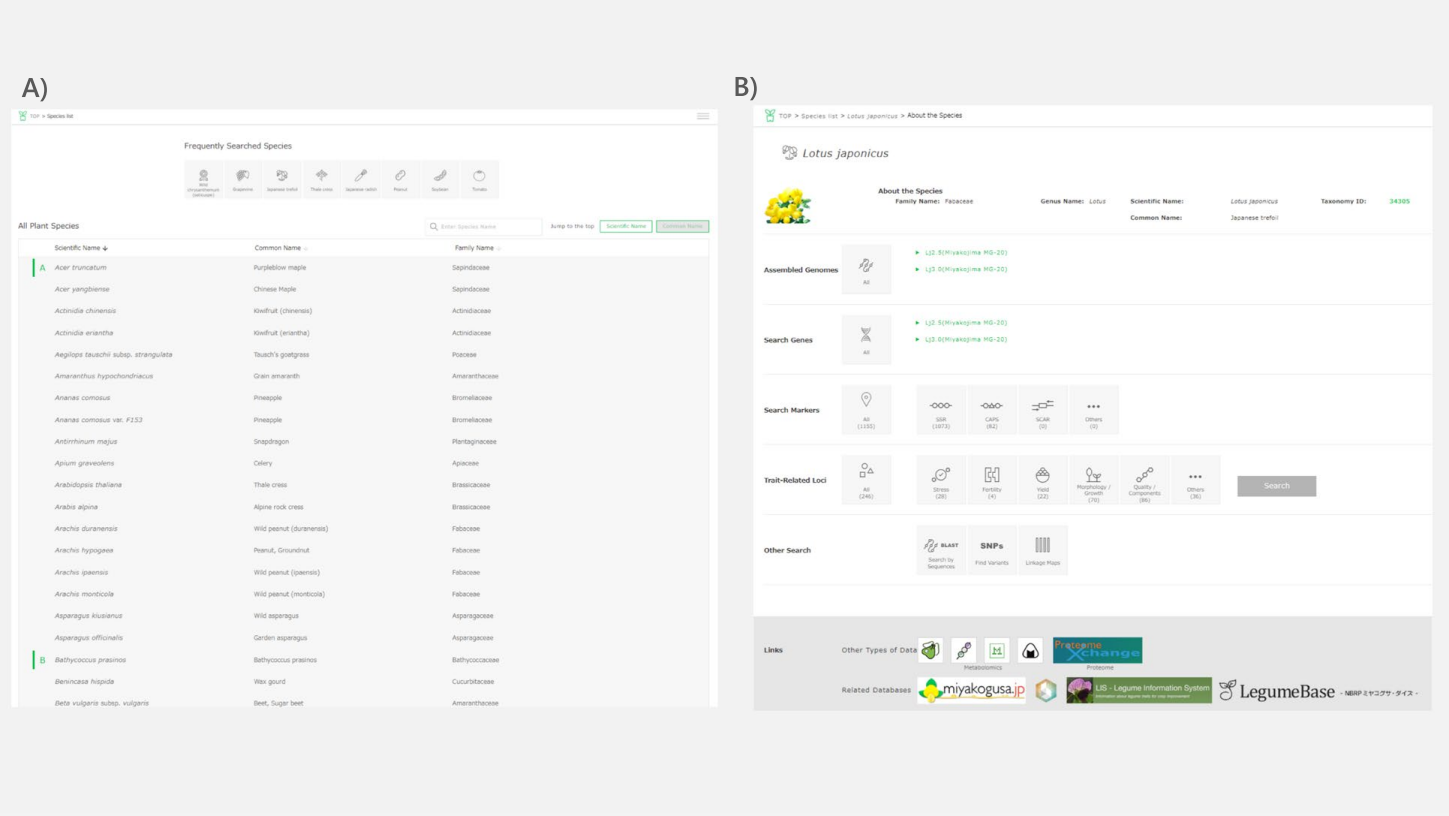
List of plant species registered in Plant GARDEN and an example of a species page. **A** Partial view of the list of plant species. A target species can be searched by scrolling the list, or by keyword search. An alphabetic search by scientific or common name is also available. **B** Species page for *Lotus japonicus*. Genome and gene sequences, markers, trait-related loci, SNPs (base variants including indels), and linkage map information are accessible on the page. Links to BLAST searches against the genomes listed on the pages and major DBs for *L. japonicus* are also shown

A list of plant species registered in Plant GARDEN is displayed by clicking on the “Search by species” button on the top page (Figs. 1 and 3A). The target species can be searched by scrolling through the list or using the keyword search window. An alphabetized search by scientific or common name can also be performed by selecting the "Sorting” arrow on the top row of the species list. Once a target species is selected from the list, a species page is shown (Fig. 3B). On the species pages, all contents described above in the “Construction and content” section are accessible. Links to BLAST searches against the genomes listed on the pages and major DBs for the species are also shown.

### Search by genes

The “search by genes” function is accessible on the top page or species pages. In cases in which users perform gene searches from the top page, all gene files registered in Plant GARDEN are targeted for search. Meanwhile, gene files in a specific species are targeted in cases where a gene search is performed using the “All” button in a gene search column on a species page. A target gene file can be chosen by selecting the genome sequence name.

**Fig. 4:**
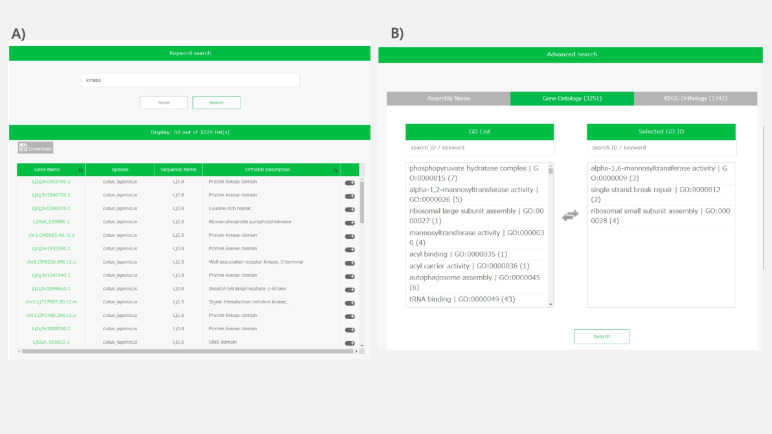
Screen view of the gene search page. **A** Example of a search of *L. japonicus* gene files by the keyword “kinase”. The hit genes are listed on the results page. **B** Screen view of an advanced search. A refined search is available by assembled genome version, gene ontology, or KEGG ontology

Two types of gene searches are available: keyword searches and refined searches. The gene keyword search targets words displayed on the gene pages (Fig. 4A). For example, all gene sequences identified on targeted genome sequences are listed when the genome sequence name is used as a keyword, because every gene sequence page displays the name of the genome sequence (e.g., Lj3.0 in Supplementary Fig. S4A). However, if the specificity of the character strings in the genome sequence name is low, gene sequences other than those on the target genome sequence may be hit, because the keyword search simply searches pages containing a target key word. Gene searches by assembled genome version, gene ontology, and KEGG ontology are available in the “advanced search” function shown under the keyword search (Fig. 4B).

### A sample data comparison by JBrowse

In Plant GARDEN, we use two genome browsers: JBrowse [14] and TASUKE + [15]. JBrowse is an excellent system for displaying and comparing diverse information and is used to visualize assembled genome sequences, gene models, DNA markers, and variants on SRA identified on the selected genome. TASUKE + is also a well-designed browser that displays multiple genomic and genetic resources, and is particularly suited to displaying variants. Therefore, TASUKE + is used to display vcf files. The data items currently displayed in JBrowse are as follows: reference sequence, gene models, markers, and base variants. At present, when multiple genomes are registered within a single species, marker and variant information is displayed only on the single genome that we have selected. For other genome sequences, only the genome sequence and gene model can be viewed on JBrowse. This is because the location information of markers or variants on the genome is determined for only the selected genomes as described in the section entitled “Base variants on SRA”.

**Fig. 5:**
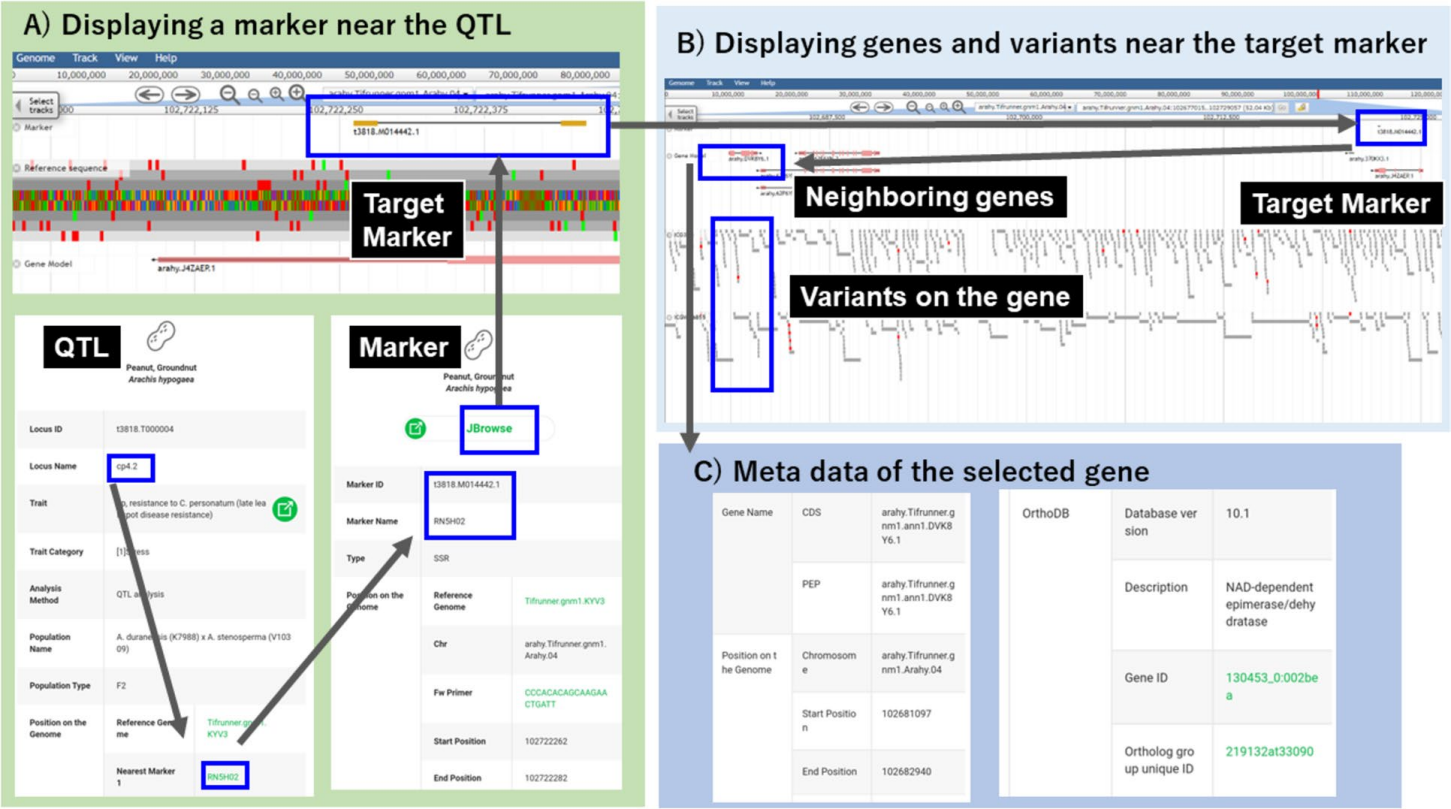
Example of the utility of JBrowse for genetic study. **A** Expanding screen view of the target marker (upper). The target marker, RN5H02 (lower right), was selected as the nearest marker of the trait-related locus, cp4.2 (lower left), controlling late leaf spot disease resistance. **B** Screen view of JBrowse with the target marker RN5H02 (the Marker ID in Plant GARDEN is t3818.M014442.1) and its neighboring genes, including arahy.DVK8Y6.1 and variants identified on the genes. **C** Screen view of the arahy.DVK8Y6.1 gene page

An example of the utility of JBrowse for genetic study is shown in Fig. 5. Leal-Bertioli et al. (2009) identified a QTL, cp4.2, related to late leaf spot resistance by using an F_2_ mapping population derived from a cross between *Arachis duranensis* and *A. stenosperma* [16]. The cp4.2 information is registered in Plant GARDEN, along with information on the nearest markers of RN5H02 and TC9E08 (Fig. 5A). The position of RN5H02 (the Marker ID in Plant GARDEN is t3818.M014442.1) on the *A. hypogaea* genome (Tifrunner.gnm1.KYV3) was determined to be around 102,722,262 bp of chromosome Arahy.04. Then genome sequences and genes near RN5H02 were confirmed on JBrowse. The nearest gene located on RN5H02, arahy.J4ZAEP.1, was annotated as an “uncharacterized protein”. However, the gene arahy.DVK8Y6.1, whose function was annotated as NAD-dependent epimerase/dehydratase in OrthDB, was found on a 41 kb upper region of RN5H02 by looking for genes on the JBrowse screen (Fig. 5B). NAD-dependent epimerase/dehydratase is considered to affect cell-surface properties [17] and was reported as an enzyme related to disease resistance in *Lens culinaris*, which belongs to *Fabaceae*, the same family to which genus *Arachis* belongs [18].

In 2009, when Leal-Bertioli et al. published their manuscript, no chromosome-level genome sequences were available. Therefore, they searched for the nearest candidate disease-resistance genes on the QTLs by mapping resistance gene analog (RGA) markers, which were designed on the nucleotide binding site domain (NBS). However, no RGA markers were determined near the cp4.2 QTL. On the other hand, Plant GARDEN makes the search for candidate genes much simpler than when Leal-Bertioli et al. (2009) published their paper. For further experiments to determine the causative mutations of the traits, the variants identified on SRA would provide useful information for identifying candidate causal variants (Fig. 5C).

### BLAST search

BLAST search pages can be accessed from the top and species pages. All sequences files registered in Plant GARDEN are listed on the BLAST search page accessed from the top page. This BLAST page can be used for cross-species searches. Meanwhile, species-specific files are listed on the BLAST search pages accessed from the species pages. The available types of searches are BLASTN, BLASTP, BLASTX, TBLASTN, and TBLASTX. The input query sequences are submitted to a server at the Kazusa DNA Research Institute, and results are returned to a Plant GARDEN page (Supplementary Fig. S8A).

In most BLAST searches, users input query sequences on a submission column by copying and pasting a query sequence from a source text. However, this operation is difficult to implement on the tablet version of the website. Therefore, the tablet version converts the text characters of a query shown in an image into text data using an OCR (Optical Character Reader). The OCR engine was developed based on Google Cloud Vision API (https://cloud.google.com/vision/). We implemented our own API that sends the acquired image data to the Google cloud and displays the obtained results on the Plant GARDEN page (Supplementary Fig. S8B).

### Tool list

Along with the widespread use of NGS technologies, the demand for self-analysis of NGS data by nonbioinformatic specialists has increased. However, many of those nonbioinformatic specialists have difficulties in setting up an analysis environment and in learning commands for analysis. Therefore, Plant GARDEN provides several analysis tools operating on its GUI (https://plantgarden.jp/en/list/tool). Two types of tools are available: those for the cloud and those for local environments. We expect that Plant GARDEN will contribute to a deeper understanding of genomic and genetic studies in various fields of expertise.

## Conclusion

Plant GARDEN is currently being developed and maintained through research funds from Kazusa DNA Research Institute as of 2023. The aim of Plant GARDEN is to provide plant genome information to the fields of plant science as well as to plant-based industries, education, and other relevant areas. Therefore, we have designed a web-based user interface (WUI) that allows a diverse range of users to access such information in an easy-to-understand manner. Plant GARDEN contains genome and gene information for 235 species as ofMarch 2023. At present, orthologous genes across species can be searched for on OrthoDB using the Ortho group unique ID associated with each gene, or by performing a BLAST search specifying multiple species sequences from the top page. Additionally, we plan to soon enable searches for orthologous genes within Plant GARDEN using the Ortho group unique IDs.

We aim to store as much genomic information in Plant GARDEN as possible. However, because of the limited amount of data we can work with, we are prioritizing data in two metrics: high-quality assembly, such as scaffolding at the chromosome level, or importance in the research community. For the latter metric, we consider two factors: high-frequency use in research communities and diversity in registered species. Because our knowledge is limited, we place importance on direct requests and opinions from users. We receive requests via e-mail (plantgarden@kazusa.or.jp) or through the questionnaire form at the Plant GARDEN site. We expect that Plant GARDEN will promote the understanding of genomes and gene diversity by facilitating comparisons of sequences registered in the DB.

### Supplementary Information

**Additional file1:** **Supplementary Fig. S1.** Structure of *pages*, the developed keyword search system. Blue and green boxes indicate external and developed search systems, respectively. Gray boxes indicate contents developed for the keyword search. **Supplementary Fig. S2.** Numbers of registered data in Plant GARDEN since 2019. The data is as of March every year. A) genome files, B) gene sequences, C) Marker. D) linkage map, E) trait related loci. Blue bars and pink lines indicate the annual registration number and cumulative number, respectively. **Supplementary Fig. S3.** A screen view of the assembled stats table on a genome sequence page of Lj3.0 *in Lotus japonicus*. We performed a test of the quality of the assembled genome and gene sequences by using BUSCOs. **Supplementary Fig. S4.** A screen view of the functional annotation in a gene sequence page, Lj3g3v0303780.2, on the Lj3.0 genome. Re annotation of all genes registered in Plant GARDEN was performed by our group using Hayai Annotation. A) A main gene table. B) A detailed table of the functional annotations displayed below the main table. The IDs shown in the detailed annotations table are linked to the external database. **Supplementary Fig. S5.** A screen view of base variant lists in *japonicus* A) A the variant call are described in the comments file. A and a text table, which show the variant bases with the letters A, G, T, and C, are available for download. The variant positions are displayed on the two browsers TASUKE + and JBrowse. B) A list of gvcf files and SRAs. The list is displayed by clicking the Line list button in the vcf file list. **Supplementary Fig. S6. **A screen view of the PCR based DNA marker lists. A) A partial view of the DNA marker list in *Vitis vinifera.* Marker names, types, and positions on the reference genome and the name of the reference genome are shown. B) The detailed information of the selected marker. The table is shown by clicking the marker ID in the marker list. The marker name, type, primer or target sequences and their positions on the genome, and source manuscript information are available. Allele information was derived from the source manuscript by manual curation. The restriction enzyme is for CAPS or dCAPS markers. **Supplementary Fig. S7.** A screen view of trait related loci lists in *Brassicarapa* A) Graphical view of registered locus positions of the reference genome. B) A part of the list of the registered trait related loci. The displayed information is changed by selecting the tabs. C) The detailed information of the selected locus. The table is shown by clicking the locus ID in the trait related loci list. The locus name, trait name, trait category, analysis method, population name, population type, position on the genome estimated via nearest markers, position on the linkage map (if available in the source manuscript), and source manuscript are shown in the table. **Supplementary Fig. S8.** BLAST analysis in Plant GARDEN. A) A screen view of BLAST analysis in the tablet version. B) The data flow of BLAST analysis in Plant GARDEN with the PC and tablet versions. Red and green represent the flow of OCR and BLAST, respectively.

## Data Availability

The database and web interface are available at https://plantgarden.jp/.

## Abbreviations

PC: Personal Computer
SNP: Single Nucleotide Polymorphism
QTL: Quantitative Trait Locus
DB: Data Base
PHP: Hypertext Preprocessor
API: Application Programming Interface
SQL: Structured Query Language
URL: Uniform Resource Locator
BUSCO: Benchmarking Universal Single-Copy Orthologs
GO: Gene Ontology
KEGG: Kyoto Encyclopedia of Genes and Genomes
EC: Enzyme Commission
TF: Transcription Factor
DDBJ: DNA Data Bank of Japan
VCF: Variant Call Format
BLAST: Basic Local Alignment Search Tool
GUI: Graphical User Interface
