# Supplementary material for: Plant GARDEN: a portal website for cross-searching between different types of genomic and genetic resources in a wide variety of plant species
Source: BMC Plant Biol. 2023 Aug 12;23:391. doi: 10.1186/s12870-023-04392-8 (PMC10422841; doi:10.1186/s12870-023-04392-8)
Supplement: Supplementary file 1 — Additional file1: Supplementary Fig. S1. Structure of pages, the developed keyword search system. Blue and green boxes indicate external and developed search systems, respectively. Gray boxes indicate contents developed for the keyword search. Supplementary Fig. S2. Numbers of registered data in Plant GARDEN since 2019. The data is as of March every year. A) genome files, B) gene sequences, C) Marker. D) linkage map, E) trait related loci. Blue bars and pink lines indicate the annual registration number and cumulative number, respectively. Supplementary Fig. S3. A screen view of the assembled stats table on a genome sequence page of Lj3.0 in Lotus japonicus. We performed a test of the quality of the assembled genome and gene sequences by using BUSCOs. Supplementary Fig. S4. A screen view of the functional annotation in a gene sequence page, Lj3g3v0303780.2, on the Lj3.0 genome. Re annotation of all genes registered in Plant GARDEN was performed by our group using Hayai Annotation. A) A main gene table. B) A detailed table of the functional annotations displayed below the main table. The IDs shown in the detailed annotations table are linked to the external database. Supplementary Fig. S5. A screen view of base variant lists in japonicus A) A the variant call are described in the comments file. A and a text table, which show the variant bases with the letters A, G, T, and C, are available for download. The variant positions are displayed on the two browsers TASUKE + and JBrowse. B) A list of gvcf files and SRAs. The list is displayed by clicking the Line list button in the vcf file list. Supplementary Fig. S6. A screen view of the PCR based DNA marker lists. A) A partial view of the DNA marker list in Vitis vinifera. Marker names, types, and positions on the reference genome and the name of the reference genome are shown. B) The detailed information of the selected marker. The table is shown by clicking the marker ID in the marker list. The marker name, type, primer [file 12870_2023_4392_MOESM1_ESM.pdf]

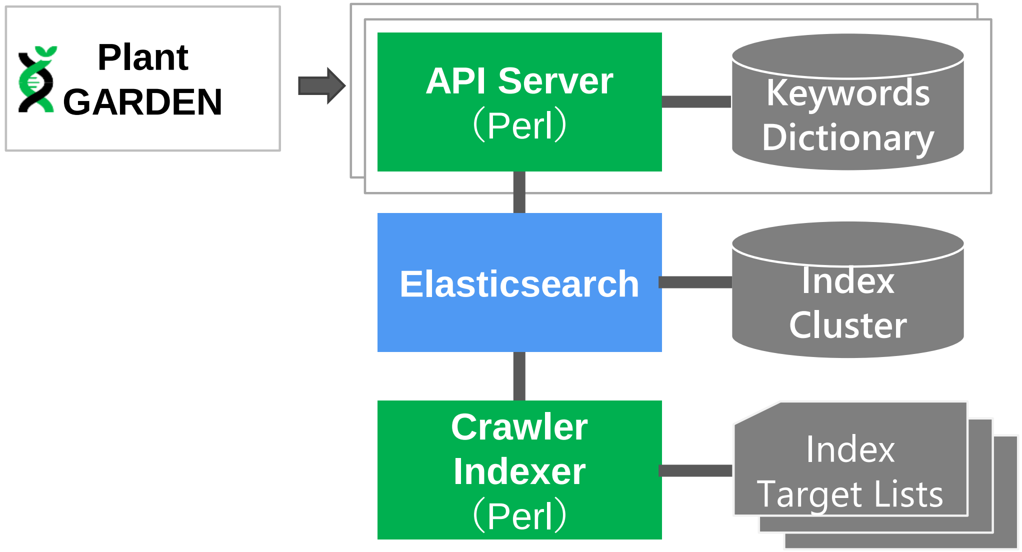

Supplementary Fig. S1.

Structure of *pages*, the developed keyword search system. Blue and green boxes indicate external and developed search systems, respectively. Gray boxes indicate contents developed for the keyword search.

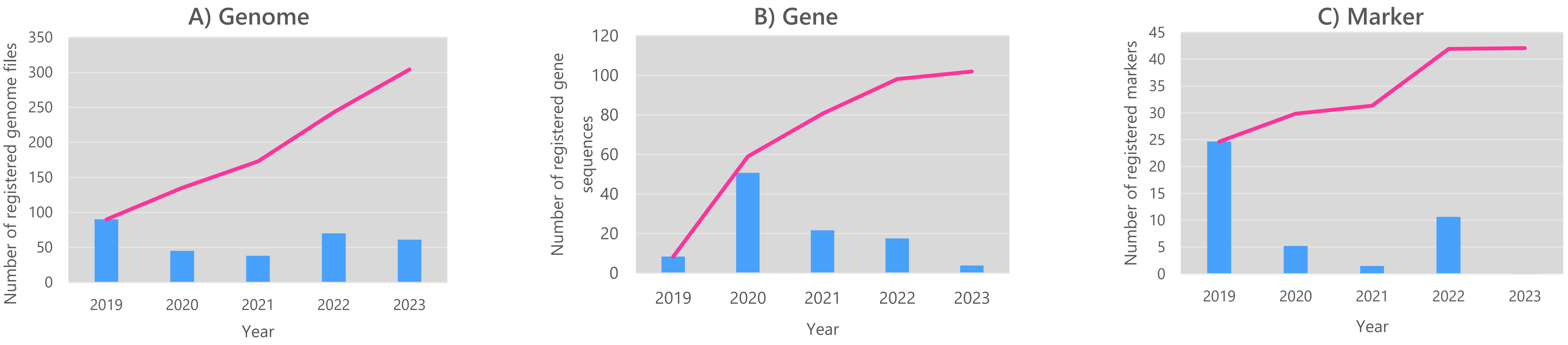

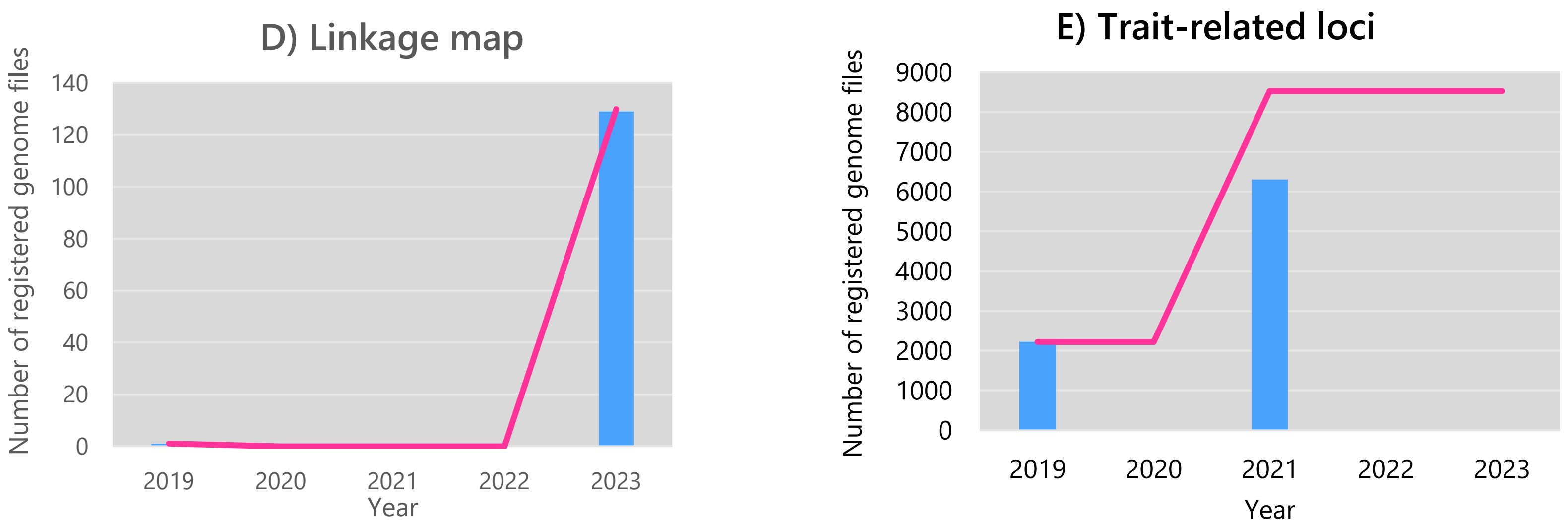

Supplementary Fig. S2.

Numbers of registered data in Plant GARDEN since 2019. The data is as of March every year. A) genome files, B) gene sequences, C) Marker. D) linkage map, E) trait related loci.

Blue bars and pink lines indicate the annual registration number and cumulative number, respectively.

Lotus japonicus

Sequence Name : Lj3.0

DOWNLOAD JBrowse

Details of the Assembled Genome Sequences			
Sequence Name	Lj3.0	Biological Sample	Miyakojima MG-20
Number of Sequences	9	Number of Chromosomes	2n = 2x = 12
Total Length (bp)	447,416,816	N50 Length (bp)	62,285,374
Number of Genes	48,105	Number of Genes Source File	Lj3.0_pep.fna
Number of Genes Annotated by Hayai-annotation	48,105	Hayai-Annotation Version	zen_v2.0
Sequencing Method	Sanger, Illumina, 454	Read Depth	35x
Assembly Method	Paracel Genome Assembler	Estimated Genome Size (Mb)	465
Sequencing Method Comments		Comments	
DOI/PMID	10.1093/dnares/dsn008	Corresponding author	Satoshi TABATA (Kazusa DNA Research Institute)
Data Source	Lotus japonicus Genome Sequencing Project	Data Source URL	http://www.kazusa.or.jp/lotus/
BUSCO Version	5.1.0	BUSCO Dataset	embryophyta_odb10
BUSCO Genome	C:93.5%[S:86.9%,D:6.6%],F:3.7%,M:2.8%,n:1614	BUSCO Gene (pep/cds)	C:79.8%[S:59.5%,D:20.3%],F:11.9%,M:8.3%,n:1614

Supplementary Fig. S3.

A screen view of the assembled stats table on a genome sequence page of Lj3.0 *in Lotus japonicus*. We performed a test of the quality of the assembled genome and gene sequences by using BUSCOs.

A)

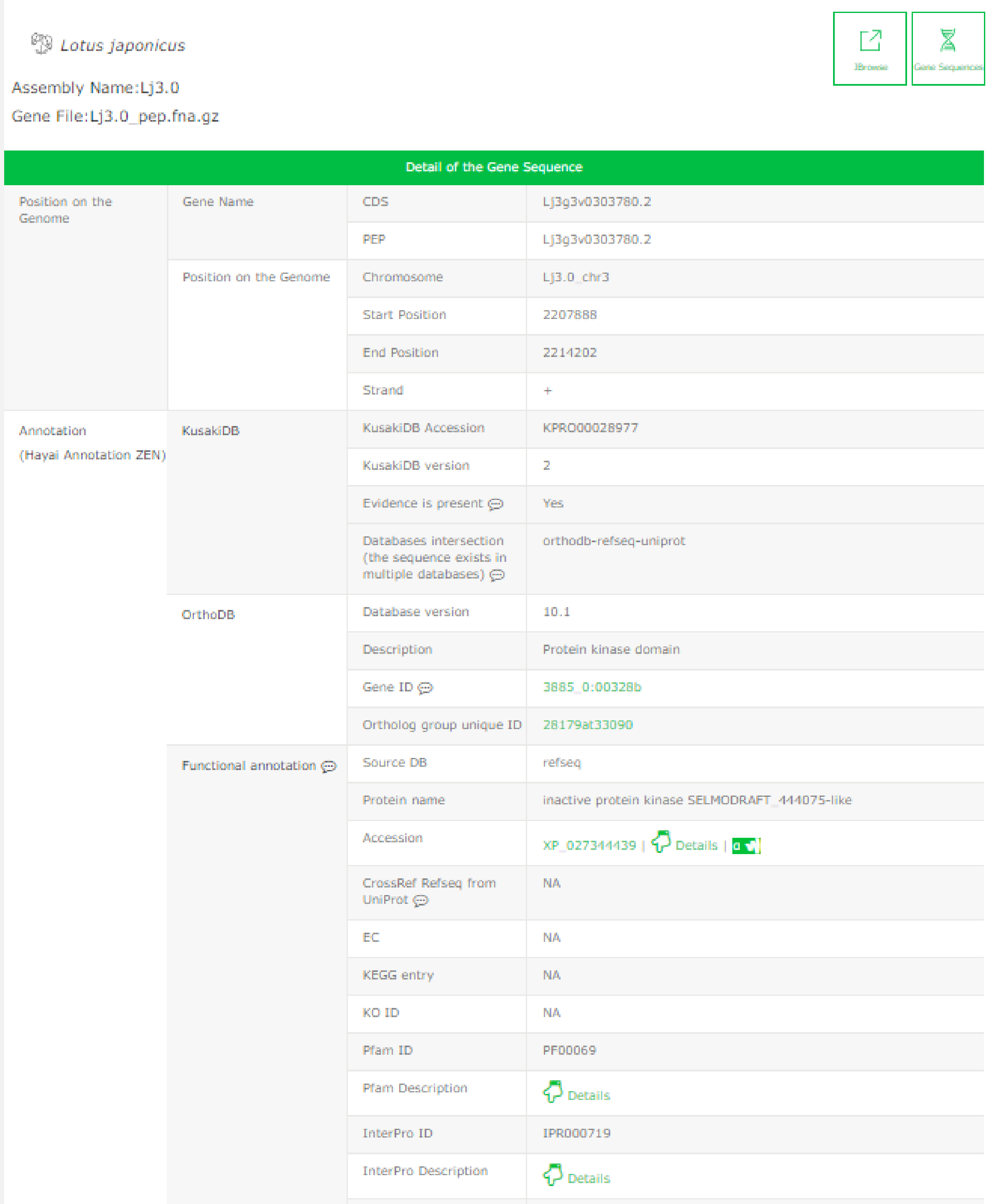

Lotus japonicus

Assembly Name:Lj3.0

Gene File:Lj3.0_pep.fna.gz

JBrowse | Gene Sequences

Detail of the Gene Sequence			
Position on the Genome	Gene Name	CDS	Lj3g3v0303780.2
		PEP	Lj3g3v0303780.2
	Position on the Genome	Chromosome	Lj3.0_chr3
		Start Position	2207888
		End Position	2214202
		Strand	+
Annotation (Hayai Annotation ZEN)	KusakiDB	KusakiDB Accession	KPRO00028977
		KusakiDB version	2
		Evidence is present	Yes
		Databases intersection (the sequence exists in multiple databases)	orthodb-refseq-uniprot
	OrthoDB	Database version	10.1
		Description	Protein kinase domain
		Gene ID	3885_0:00328b
		Ortholog group unique ID	28179at33090
	Functional annotation	Source DB	refseq
		Protein name	inactive protein kinase SELMODRAFT_444075-like
		Accession	XP_027344439 | Details |
		CrossRef Refseq from UniProt	NA
		EC	NA
		KEGG entry	NA
		KO ID	NA
		Pfam ID	PF00069
		Pfam Description	Details
		InterPro ID	IPR000719
		InterPro Description	Details

B)

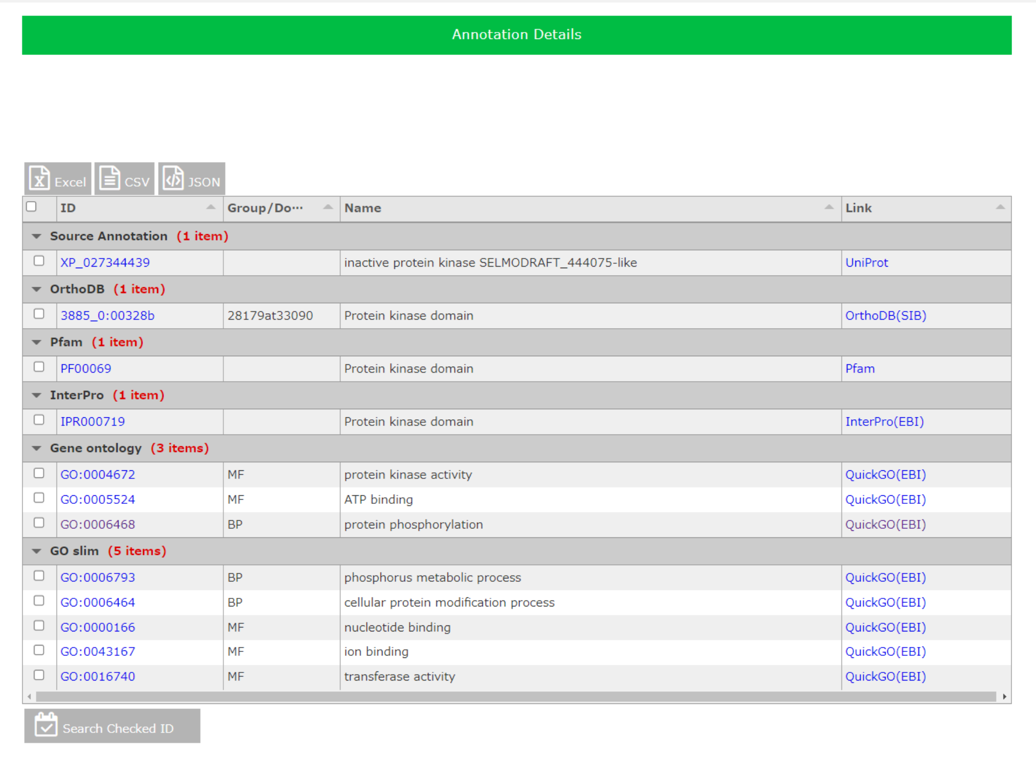

Annotation Details

Excel | CSV | JSON

ID	Group/Do···	Name	Link
**Source Annotation (1 item)**			
XP_027344439		inactive protein kinase SELMODRAFT_444075-like	UniProt
**OrthoDB (1 item)**			
3885_0:00328b	28179at33090	Protein kinase domain	OrthoDB(SIB)
**Pfam (1 item)**			
PF00069		Protein kinase domain	Pfam
**InterPro (1 item)**			
IPR000719		Protein kinase domain	InterPro(EBI)
**Gene ontology (3 items)**			
GO:0004672	MF	protein kinase activity	QuickGO(EBI)
GO:0005524	MF	ATP binding	QuickGO(EBI)
GO:0006468	BP	protein phosphorylation	QuickGO(EBI)
**GO slim (5 items)**			
GO:0006793	BP	phosphorus metabolic process	QuickGO(EBI)
GO:0006464	BP	cellular protein modification process	QuickGO(EBI)
GO:0000166	MF	nucleotide binding	QuickGO(EBI)
GO:0043167	MF	ion binding	QuickGO(EBI)
GO:0016740	MF	transferase activity	QuickGO(EBI)

Search Checked ID

Supplementary Fig. S4.
A screen view of the functional annotation in a gene sequence page, Lj3g3v0303780.2, on the Lj3.0 genome. Re-annotation of all genes registered in Plant GARDEN was performed by our group using Hayai-Annotation. A) A main gene table. B) A detailed table of the functional annotations displayed below the main table. The IDs shown in the detailed annotations table are linked to the external database.

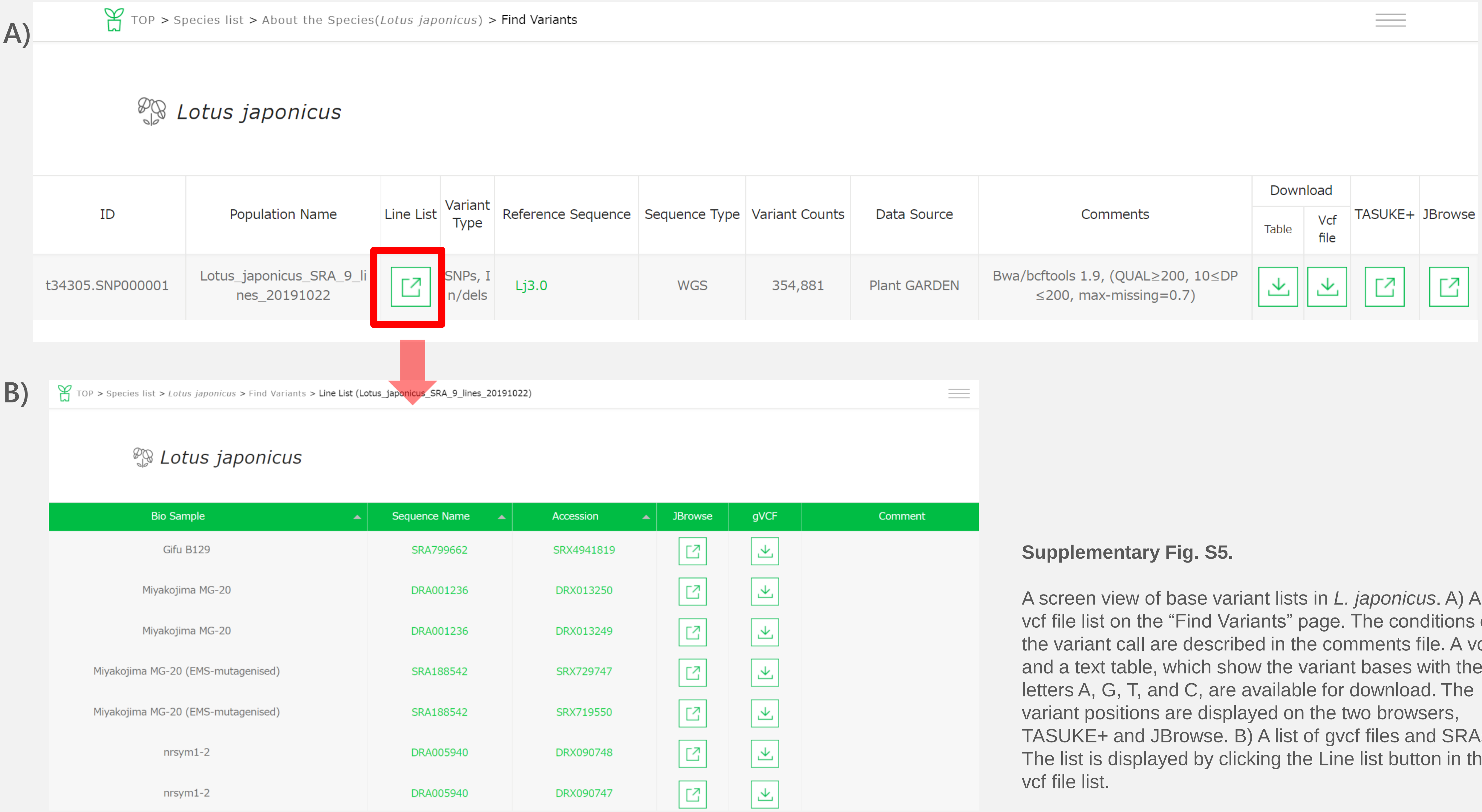

Supplementary Fig. S5.

A screen view of base variant lists in *L. japonicus*. A) A vcf file list on the "Find Variants" page. The conditions of the variant call are described in the comments file. A vcf and a text table, which show the variant bases with the letters A, G, T, and C, are available for download. The variant positions are displayed on the two browsers, TASUKE+ and JBrowse. B) A list of gvcf files and SRAs. The list is displayed by clicking the Line list button in the vcf file list.

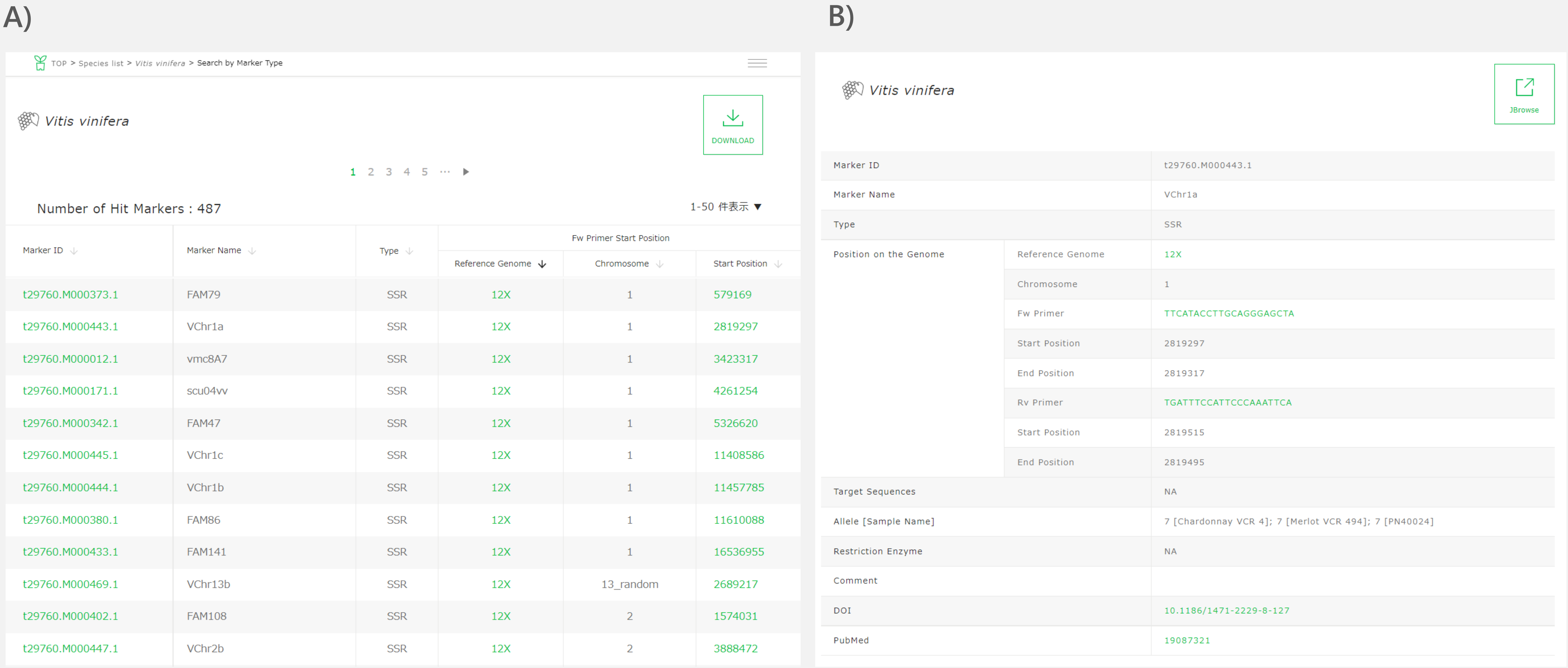
A)

TOP > Species list > *Vitis vinifera* > Search by Marker Type

Vitis vinifera

DOWNLOAD

1 2 3 4 5 ⋯ ▶

Number of Hit Markers : 487

1-50 件表示 ▼

Marker ID	Marker Name	Type	Fw Primer Start Position		
			Reference Genome	Chromosome	Start Position
t29760.M000373.1	FAM79	SSR	12X	1	579169
t29760.M000443.1	VChr1a	SSR	12X	1	2819297
t29760.M000012.1	vmc8A7	SSR	12X	1	3423317
t29760.M000171.1	scu04vv	SSR	12X	1	4261254
t29760.M000342.1	FAM47	SSR	12X	1	5326620
t29760.M000445.1	VChr1c	SSR	12X	1	11408586
t29760.M000444.1	VChr1b	SSR	12X	1	11457785
t29760.M000380.1	FAM86	SSR	12X	1	11610088
t29760.M000433.1	FAM141	SSR	12X	1	16536955
t29760.M000469.1	VChr13b	SSR	12X	13_random	2689217
t29760.M000402.1	FAM108	SSR	12X	2	1574031
t29760.M000447.1	VChr2b	SSR	12X	2	3888472

B)

Vitis vinifera

JBrowse

Marker ID		t29760.M000443.1
Marker Name		VChr1a
Type		SSR
Position on the Genome	Reference Genome	12X
	Chromosome	1
	Fw Primer	TTCATACCTTGCAGGGAGCTA
	Start Position	2819297
	End Position	2819317
	Rv Primer	TGATTTCCATTCCCAAATTCA
	Start Position	2819515
	End Position	2819495
Target Sequences		NA
Allele [Sample Name]		7 [Chardonnay VCR 4]; 7 [Merlot VCR 494]; 7 [PN40024]
Restriction Enzyme		NA
Comment		
DOI		10.1186/1471-2229-8-127
PubMed		19087321

Supplementary Fig. S6.

A screen view of the PCR-based DNA marker lists. A) A partial view of the DNA marker list in *Vitis vinifera*. Marker names, types, and positions on the reference genome and the name of the reference genome are shown. B) The detailed information of the selected marker. The table is shown by clicking the marker ID in the marker list. The marker name, type, primer or target sequences and their positions on the genome, and source manuscript information are available. Allele information was derived from the source manuscript by manual curation. The restriction enzyme is for CAPS or dCAPS markers.

A)

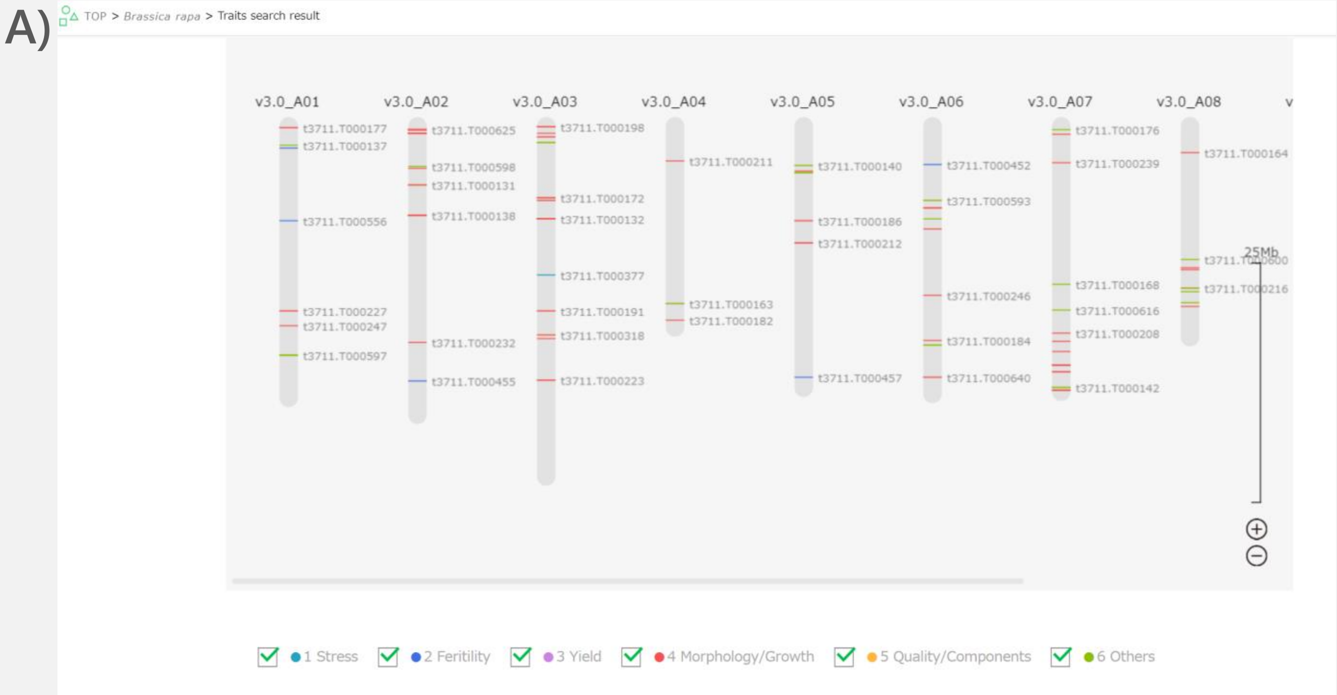

B)

Brassica rapa

DOWNLOAD

1 2 3 4 5 ··· ▸

Results : 642 Hit(s)

Trait	Category	Nearest Marker 1	Nearest Marker 2	Trait / Analysis Method / Population Name / Population Type		Other Data	Locus ID
		Reference Genome	Nearest Marker 1	Chromosome	Start Position	End Position	
Bolting time	Morphology/Growth	BrapaV3.0	cnu_m616a	A02	721260	721528	t3711.T000124
Bolting time	Morphology/Growth	BrapaV3.0	cnu_m335a	A08	19606371	19606574	t3711.T000125
Bolting time	Morphology/Growth	BrapaV3.0	cnu_m372a	A09	34123484	34123659	t3711.T000126
Bolting time	Morphology/Growth	BrapaV3.0	ACMP00598	A10	15798743	15799010	t3711.T000127
Bolting time	Morphology/Growth	BrapaV3.0	cnu_m056a	A07	26030383	26030580	t3711.T000128
Bolting time	Morphology/Growth	BrapaV3.0	SNP-CKX3a	A02	6286170	6286372	t3711.T000131
Bolting time	Morphology/Growth	BrapaV3.0	cnu_m288a	A03	9986707	9986860	t3711.T000132

C)

Locus ID		t3711.T000124
Locus Name		qDB1
Trait		Bolting time
Trait Category		[4]Morphology/Growth
Analysis Method		QTL analysis
Population Name		CRF3
Population Type		F2:3
Position on the Genome	Reference Genome	BrapaV3.0
	Nearest Marker 1	cnu_m616a
	Chromosome	A02
	Start Position	721260
	End Position	721528
	Nearest Marker 2	At5g12290
	Chromosome	NA
	Start Position	0
	End Position	0
Position(cM)	Linkage Group	CRF3 ChrA02
	LOD Peak Position(cM)	0
	Max LOD	4.8
	P Value	
DOI/PMID		10.1093/dnares/dss029
Comment		Chiifu 401-42:long flowering time after vernalization, high plant height, large leaf, many seeds per siliqua, better seed weight; RCBr:rapid cycling, short flowering time after vernalization, low plant height, small leaf, low seeds per siliqua, lower seed weight

Supplementary Fig. S7.

A screen view of trait-related loci lists in *Brassica rapa*. A) Graphical view of registered locus positions of the reference genome. B) A part of the list of the registered trait-related loci. The displayed information is changed by selecting the tabs. C) The detailed information of the selected locus. The table is shown by clicking the locus ID in the trait-related loci list. The locus name, trait name, trait category, analysis method, population name, population type, position on the genome estimated via nearest markers, position on the linkage map (if available in the source manuscript), and source manuscript are shown in the table.

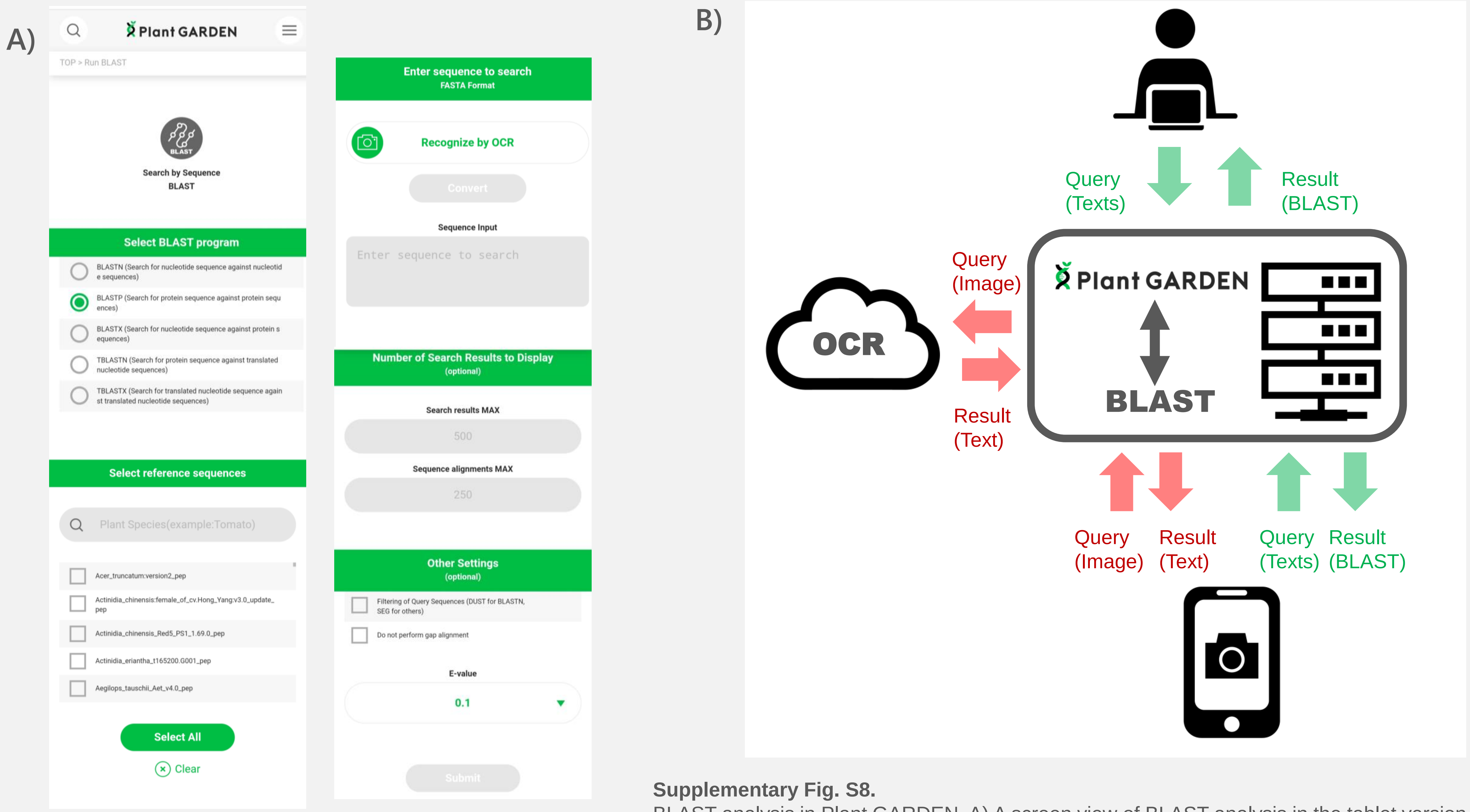

Supplementary Fig. S8.
BLAST analysis in Plant GARDEN. A) A screen view of BLAST analysis in the tablet version. B) The data flow of BLAST analysis in Plant GARDEN with the PC and tablet versions. Red and green represent the flow of OCR and BLAST, respectively.
